# Heat shock protein 72 expression allows permissive replication of oncolytic adenovirus dl1520 (ONYX-015) in rat glioblastoma cells

**DOI:** 10.1186/1476-4598-4-12

**Published:** 2005-03-11

**Authors:** Jonathan Madara, James A Krewet, Maulik Shah

**Affiliations:** 1 Saint Louis University Cancer Center, Saint Louis University, USA; 2 School of Medicine, Saint Louis University, USA; 3 Center for Anatomic Studies, Saint Louis University, USA; 4 Department of Pediatrics, Division of Medical Genetics, Saint Louis University, USA

## Abstract

In this study we have made novel observations with regards to potentiation of the tumoricidal activity of the oncolytic adenovirus, dl1520 (ONYX-015) in rat glioblastoma cell lines expressing heat shock protein 72 (HSP72) due to permissive virus replication. ONYX-015 is a conditionally replicating adenovirus that is deleted for the E1B 55 kDA gene product whose normal function is to interact with cell-cycle regulatory proteins to permit virus replication. However, many murine and rodent cell lines are not permissive for adenovirus replication. Previously, it has been reported that the heat shock response is necessary for adenovirus replication and that induction of heat shock proteins is mediated by E1 region gene products. Therefore, we hypothesized that HSP72 expression may allow for permissive replication of ONYX-015 in previously non-permissive cells. Rat glioma cell lines 9L and RT2 were transfected with a plasmids expressing HSP72 or GFP. After infection with ONYX-015, no tumoricidal activity is observed in GFP expressing cell lines despite adequate transduction. In contrast, HSP72 transfected cells show cytopathic effects by 72 hours and greater than 75% loss of viability by 96 hours. Burst assays show active virus replication in the HSP72 expressing cell lines. Therefore, 9L-HSP72 and RT2-HSP72 are ideal models to evaluate the efficacy of ONYX-015 in an immunocompetent rat model. Our study has implications for creating rodent tumor models for pre-clinical studies with E1 region deleted conditionally replicating adenovirus.

## Background

Adenovirus vectors are commonly utilized in cancer gene therapy experiments. They readily infect numerous tumor cell types and are easily manipulated allowing for transgene expression[1]. In an effort to improve selectivity of these vectors for malignant cells, replication selective or conditionally replicating adenoviruses were created[2]. With greater understanding in the molecular aspects of adenovirus replication, these viruses were designed such that replication was predicated on alterations in cell cycle regulation, thus rendering only malignant cells susceptible. These adenovirus systems rely on virus replication as a means of exerting tumoricidal effect. One of the first of these conditionally replicating adenoviruses was dl1520 or ONYX-015[3]. It has been used extensively in cancer gene therapy clinical trials[4,5]. This virus is deleted for the E1B-55 kDA gene[6].

The E1 region genes of the common serotypes of adenovirus optimize their own replication in target cells and interact with many cell cycle associated gene products. The E1a region genes promote transition of cells into S phase through their interaction with the retinoblastoma protein (pRB)[7]. pRB in conjunction with p53 can inhibit transition of cells into the S-phase of the cell cycle. Binding of E1a to pRB prevents association of pRB to the E2F transcription factor resulting in activation of E2F[8]. E2F then functions to transition cells into the S-phase of the cell cycle which is conducive for optimal adenovirus replication. In wild type adenoviruses, the role of the E1B-55 kDa gene product is to neutralize p53[9,10]. p53 induction is thought to promote cell cycle arrest resulting in termination of the virus replicative lifecycle in that cell[11]. Thus it was hypothesized that deletion of the E1B-55 kDa gene product would result in p53 induced termination of virus replication in normal cells while being replication permissive in malignant cells with abnormal p53 expression or regulation[9]. Early reports with ONYX-015 indicated replication selectivity for tumor cells with abnormal p53 functioning[12]. However, the exact mechanism of replication selectivity has been brought to question with subsequent reports of ONYX-015 replication independent of p53[13] and researchers have looked at other factors associated with adenovirus replication.

Various heat shock proteins have been shown to be necessary for efficient adenovirus replication. The avian adenovirus CELO requires the induction of HSP70 and HSP40 for production of viral proteins[14]. CELO mutants lacking the E1 region genes were replication incompetent in A549 cells. However, heat shock protein induction or expression allowed for permissive replication of these mutants. Similarly in a number of human cell lines, both heat shock and HSP72 expression enhanced the oncolytic effect of E1 containing adenovirus but not for E1 deleted adenovirus[15].

Adenovirus E1 region genes from serotype 5 and 12 have previously been shown to induce HSP72 expression [16]. HSP72 is a molecular chaperone protein involved in the repairing denatured proteins. Through this role, it can inhibit apoptosis downstream of caspase activation but prior to loss of mitochondrial membrane potential[17]. Stably transfected or E1 transformed cell lines show constitutive expression of the inducible HSP72 [18]. HSP72 levels are correlated with cell-cycle with increases in HSP72 during S-phase with a maximum level in the post-S-phase period [19]. In 293 cells which are transformed with the adenovirus E1 region gene, E1A mRNA accumulated just prior to HSP72 expression suggesting that E1A is responsible for the cell cycle regulation of HSP72 expression [18]. Additionally, HSP72 co-localizes with E1A gene products in infected cells [20]. HSP72 is predominately cytoplasmic but translocates to the nucleus following adenovirus infection. Double labelling experiments with E1A and HSP72 in infected cells showed co-localization of these molecules within the nucleoli and exhibited similar reticular and punctuate nuclear staining patterns depending on cell cycle. HSP72 transport to the nucleus was E1A and virus infection dependent. In 293 (E1 region transformed) cells, HSP72 and E1A co-localization was not observed until after infection with virus [20]. These data suggest a physical complex is necessary between adenovirus E1A, HSP72 and other adenovirus gene products

One of the difficulties with studying E1 adenovirus deletion mutants is the lack of appropriate animal tumor models which allow for permissive growth of these viruses. Common animal tumor models for glioblastoma are the cell lines 9L and RT2. Previously, it has been shown that replication competent E1 deletion mutants can transduce 9L cells and result in transgene expression but there is a block against virus replication[21]. In this study we addressed if HSP72 expression could allow for permissive replication of the ONYX-015 adenovirus in these rat glioblastoma cells.

## Results

### Transduction Efficiency

Prior to studying the efficacy of ONYX-015 to result in tumor cell lysis, we determined the optimal dose necessary for tissue transduction. Adenovirus infection efficiency is predicated on the expression of adenovirus surface receptors[22]. Using a recombinant adenovirus expressing green fluorescent protein (GFP), we transduced various glioma cell lines and analyzed them by fluorescence microscopy. Results are summarized in Table 1. Both the rat cell lines, 9L and RT2 required a significantly high dose of adenovirus to result in effective transduction. In contrast, the human glioma cell lines, DBTRG and NB4, require much less virus for efficient transduction.

**Table 1:** Transduction efficiency of various CNS tumor cell lines by AD-GFP Numbers represent percentage of cells transduced with a recombinant adenovirus vector expressing green fluorescent protein (AD-GFP) at the stated multiplicity of infection (MOI). Transduction was determined by fluorescence microscopy of cells on a hemacytometer and is represented as % of total cells.

	**Multiplicity of Infection**						
	
**Cell Line**	**0**	**1**	**10**	**100**	**300**	**500**	**1000**
	
9L	0	0	1	27	54	63	74
RT2	0	1	8	41	68	81	93
DBTRG	0	69	100	100	-	-	-
NB4	0	74	100	100	-	-	-

### Cytopathic Effect

**Figure 1:**
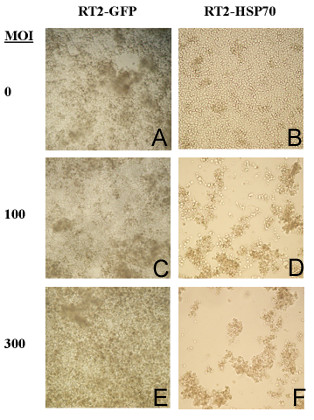
**Cytopathic effect of ONYX-015 on GFP and HSP72 transfected glioma cells. **Photographs (100× magnification) of RT2-GFP and RT2-HSP72 cells 96 hours after ONYX-015 infection at various dosages are represented. A – RT2-GFP cells mock infected with virus. B – RT2-HSP72 cells mock infected with virus. C – RT2-GFP cells infected at MOI of 100. D – RT2-HSP72 cells infected at MOI of 100. E – RT2-GFP cells infected at MOI of 300. F – RT2-HSP72 cells infected at MOI of 300.

The dramatic difference in tumoricidal effect between HSP72 transfected and control tumor cells is best observed by cytopathic effect. In Figure 1 we show the affects of virus infection on RT2-GFP and RT2-HSP72 cells 96 hours after infection with ONYX-015. Non-infected RT2-GFP and RT2-HSP72 cells showed normal glioma histological features and growth characteristics (Figure 1, panel A and B). The cells were infected when 50% confluent. At 96 hours, RT2-GFP cells infected at MOI of 100 or 300 showed little cytopathic effect and very little difference was ascertained in comparison to non-infected cells (Figure 1, panel C and E). In contrast, the majority of RT2-HSP72 cells were dead or dying (Figure 1, panel D and F). The few remaining cells were larger in size with oval or round morphology. These cells showed morphological characteristics consistent with adenovirus replication as observed in 293 cells. Similar results were obtained when assessing 9L-HSP72 cells. In table 2 we show the time to full CPE in the rat glioblastoma cell lines and a variety of human controls. Given the differences in transduction efficiency, the human cell lines were infected at a lower multiplicity of infection. No CPE is detected in the 9L-GFP and RT2-GFP cell lines. In contrast, 9L-HSP72 and RT2-HSP72 show CPE in as little as 4 days comparable to human glioblastoma cells when infected at an MOI of 10 and show CPE more rapidly than the human cell lines despite a lesser transduction efficiency.

**Table 2:** Time to Cytopathic Effect Cells grown in 96 well plates were infected with ONYX-015 at the stated multiplicity of infection per row of cells. Values represent days until 50% of wells in a row showed cytopathic effect.

	**Multiplicity of Infection**				
	
**Cell Line**	**0**	**1**	**10**	**100**	**300**
	
9L-GFP	0	0	0	0	0
9L-HSP72	0	0	8	5	4
RT2-GFP	0	0	0	0	0
RT2-HSP72	0	0	0	4	4
DBTRG	0	6	4	-	-
NB4	0	5	4	-	-
293	0	2	1	-	-

### HSP72 Expression Augments ONYX-015 Toxicity

**Figure 2:**
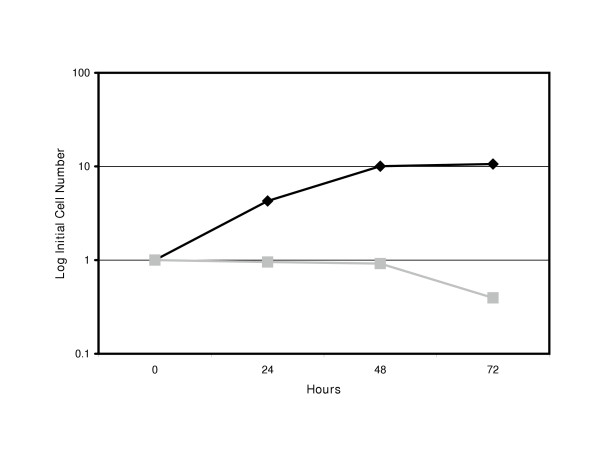
**Cytostatic effect of ONYX-015 infection on HSP72 transfected 9L cells. **9L-HSP72 cells were transduced with either AD-GFP (□) or ONYX-015 (■) at a multiplicity of infection of 100. Cell numbers were determined over time (X-axis) by direct counting using a Coulter Counter. Values (Y-axis) represent log of cell number as a fraction of the initial cell number plated.

When HSP72-transfected cells were infected with a low dose (MOI 100) of ONYX-015, there is no loss of cell viability. However, comparison of cell numbers to GFP-transfected cells or AD-GFP infected cells showed a dramatic cytostatic effect (Figure 2). 9L-GFP and 9L-HSP72 cells were transduced with either AD-GFP or ONYX-015. There is no effect on cell proliferation over time of the 9L-GFP cells infected with either AD-GFP or ONYX-015 (data not presented). In contrast, the 9L-HSP72 cells show normal proliferation when infected with AD-GFP infected cells and their cell numbers increase over time. However, ONYX-015 infected 9L-HSP72 cells showed loss of proliferative potential with very little increase in cell number over time. After 72 hours, the AD-GFP infected cells showed a decrease in proliferation but this is likely due to overcrowding of cells in the tissue culture vessel and/or the rapid utilization of consumable energy in the media.

**Figure 3:**
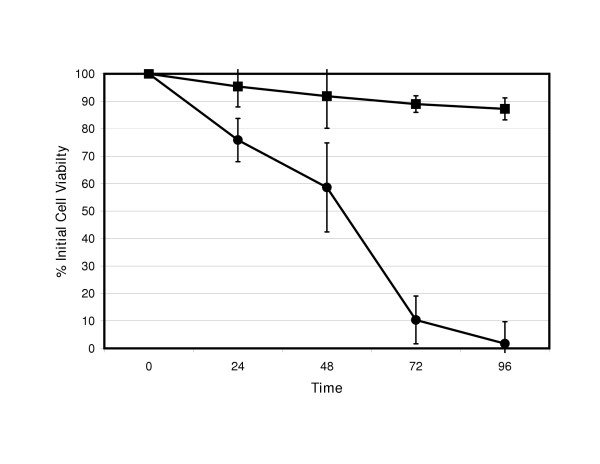
**9L Cell viability after ONYX-015 infection. **Cell viability (as % viable cells; y-axis) of 9L-GFP (■) and 9L-HSP72 (●) cell lines after infection with ONYX-015 at a MOI of 300 over time (x-axis). There is statistical significance (p < 0.05) when comparing GFP and HSP72 transfected cell numbers by analysis of variance at the 72 and 96 hour time points.

**Figure 4:**
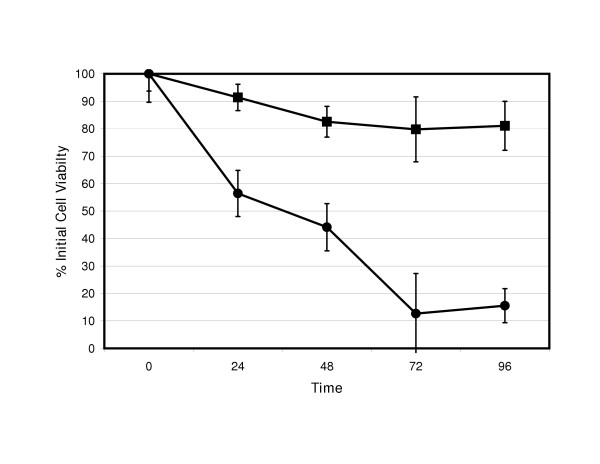
**RT2 Cell viability after ONYX-015 infection. **Cell viability (as % viable cells; y-axis) of RT2-GFP (■) and RT2-HSP72 (●) cell lines after infection with ONYX-015 at a MOI of 300 over time (x-axis). There is statistical significance (p < 0.05) when comparing GFP and HSP72 transfected cell numbers by analysis of variance at the 72 and 96 hour time points.

When assessing for cell viability, 9L-GFP (figure 3) and RT2-GFP (figure 4) cells exhibited minimal toxicity when infected with ONYX-015. In contrast, both 9L-HSP72 and RT2-HSP72 cell lines were more susceptible to ONYX-015 cell lysis. In both HSP72 transfected cell lines, loss of cell viability began as early as 24 hours after infection. Both 9L-HSP72 and RT2-HSP72 show greater than 90% loss of viability by 96 hours.

### Burst Assays

**Figure 5:**
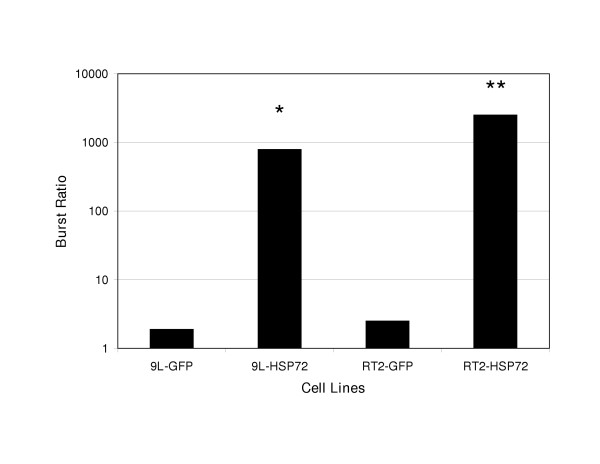
**Burst assays. **Burst ratio is given as the virus titer at 72 hours compared to 4 hours of various cell lines (x-axis) infected while 50% confluent with ONYX-015 at MOI of 100. Results are representative of a typical experiment of at least three performed. (*) represents statistical significance (p < 0.05) by Student's t-test between 9L-HSP72 and 9L-GFP. (**) represents statistical significance (p < 0.05) by Student's t-test between RT2-HSP72 and RT2-GFP.

The mechanism of cell death associated with replication competent adenoviruses has been attributed to release of virus from cells following virus replication. Having observed cytopathic effects (figure 1) in association with ONYX-015 infection of 9L-HSP72 and RT2-HSP72 cells, we presumed the same mechanism applied. To confirm this hypothesis we evaluated adenovirus replication in GFP and HSP72 transfected tumor cells by burst assay. In a normal burst assay, an increase in virus titer after 72 hours is indicative of virus replication. Tumor cells were also infected with the replication incompetent virus (AD-GFP) as a negative control and no significant virus replication was measured (data not presented). Since HSP72 transfected cells were susceptible to ONYX-015 mediated cell death we expected an increase in virus titer if the cell death was a result of permissive adenovirus replication. After infection of cells with ONYX-015, the 9L-HSP72 and RT2-HSP72 cells showed an increase in virus titer confirming virus replication (figure 5) in contrast to 9L-GFP and RT2-HSP72 transfected cells. There was a direct correlation between virus titer and cell death. Virus titer in both GFP transfected and HSP72 transfected cells increased in proportion to cell death.

## Discussion

ONYX-015 is a promising new cancer gene therapy vector which exerts its tumoricidal effect by selective replication in cancer cells[6]. Replication selectivity previously was attributed to interactions of E1 gene products with p53[3]. Subsequent reports have shown that ONYX-015 replicates in cells with wild-type p53 as efficiently as in cells with mutant-p53[13]. Although the mechanism of cancer cell selectivity is still under investigation, the various hypotheses consistently involve cell-cycle regulatory proteins and their interactions within apoptosis pathways. HSP72 is a potent regulator of apoptosis [23]. Also, there is evidence that activation of the heat shock response by adenovirus early region genes is necessary for virus replication[14]. Previously it has been shown that heat shock induced HSP72 expression resulted in increased production of adenovirus proteins[24]. Additionally, the E1A adenovirus gene products necessary for virus genome replication have previously been shown to co-localize with HSP72[20].

To study the role of HSP72 in augmenting ONYX-015 replication, we chose two glioma cell lines with different derivations. 9L is a chemically induced gliosarcoma while RT2 is a virally transformed glioblastoma. Compared to many human tumor cell lines, these cell lines show relative resistance to the cytotoxic affects of ONYX-015. Resistance in part was secondary to low adenovirus transduction efficiency. At the multiplicities of infection utilized in this study, we never achieved 100% cell transduction (table 1). In most cases, when less than 30% of permissive HSP72-transfected cells were transduced, tumor cell growth was rapid and outpaced ONYX-015 virus replication resulting in no discernable cytotoxic or cytostatic effect after 1 week in culture (data not presented). Therefore, replicative adenovirus cytotoxicity is likely a balance between virus transduction efficiency, replication efficiency and cell growth characteristics. HSP72 transfection had no bearing on cell doubling time of either 9L or RT2 cells (data not presented) RT2 cell-doubling time is approximately 8 hours while 9L cell-doubling time is approximately 14 hours. Both cell lines after HSP72 transfection showed equal susceptibility to ONYX-015. Further evidence for the balance between transduction efficiency, replication efficiency and cell doubling time is evident in that ONYX-015 infection resulted in a predominantly cytostatic affect resulting in decreased cell division before evidence of cytotoxicity.

The most dramatic affect of the role HSP72 in promotion of virus replication is evident in figure 1. At 96 hours, the GFP transduced cells showed very little cytotoxic effect of ONYX-015 infection. In contrast, there is a distinct cytopathic effect at the same doses of ONYX-015 infection in the HSP72 transduced cells. These cells were rendered susceptible to ONYX-015 toxicity. The mechanism of cell death is related to increased virus replication. Significantly more ONYX-015 was isolated from HSP72 transduced cells than the GFP-transfected controls.

The E1a gene products of adenovirus are responsible for activation of the HSP72 promoter and may affect HSP72 levels during the cell cycle[18]. Conversely, there is no evidence that HSP72 interacts with adenovirus promoters to stimulate viral transcription. There is evidence of HSP72 interactions with adenovirus structural proteins such as hexon[25] and fiber[26]. Adenovirus assembly is known to be inefficient with only a small percentage of total structural proteins eventually utilized in the production of infectious virus [26].

In conclusion, our studies demonstrated that HSP72 expression in rodent glioma tumor cells potentiated ONYX-015 replication and oncolysis of tumor cells. Further studies on the role of HSP72 in tumor types with wild-type and mutant p53 tumor suppressor genes is warranted to understand the molecular interactions of this chaperone protein in promoting virus replication. Additionally, addressing the role of HSP72 as an inhibitor of apoptosis in virus replication would further help characterize the interaction of cell-cycle regulatory pathways with virus replication. Furthermore, we intend to evaluate the role of HSP72 associated permissive replication in other animal cell lines to establish animal models for the study of ONYX-015 and similar replication competent adenoviruses.

## Conclusion

For clinical applicability, these findings have a number of implications. Currently, clinical trials are ongoing with ONYX-015. Due to the previous association of ONYX-015 replication with p53 status, the current tumor types chosen for clinical trials are those exhibiting abnormal p53 expression in a majority of cells. Our study indicates, that tumor types with high HSP72 may also be viable candidates to evaluate ONYX-015 efficacy, as they are likely to demonstrate enhanced susceptibility to this replication competent virus. As HSP72 expression in tumors is associated with a more aggressive tumor phenotype, ONYX-015 may be ideal as adjunctive therapy for advanced disease. Additionally, one of the greatest limitations to adenovirus gene therapy is patient safety at high doses of adenovirus administration. Since, HSP72 expression resulted in oncolysis at much lower MOIs, a lower dose of virus administered to a HSP72 positive tumor may have the same benefit as a higher dose, allowing one to use lower doses to achieve the same effect. In addition, for some tumor types such as breast cancer, hyperthermia has been shown to significantly improve response rates[27]. HSP72 is readily induced with hyperthermia. Therefore, HSP72 induction by hyperthermia may also serve as an effective strategy to augment ONYX-015 oncolytic activity. We are currently in the process of studying this hypothesis. Lastly, the role of HSP72 in cell cycle regulation suggests alternate pathways for the mechanism of E1b deleted adenovirus replication in tumorigenic cells. HSP72 overexpression in non-transformed fibroblasts did not result in ONYX-015 oncolysis (data not presented). Understanding the molecular interactions of adenovirus proteins with molecular chaperones would help determine cell selectivity to replication competent adenoviruses influencing the design of more potent viruses.

## Methods

### Cell Lines

9L and RT2 cells are rat glioma cell lines with different derivations. 9L cells are chemically induced gliosarcoma cells[28] while RT2 are virally transformed and have glioblastoma histology[29]. These cell lines were obtained from Dr. Martin Graf (Medical College of Virginia, Richmond, VA). Cells were kept at 37°C, 5% CO_2 _and 95% humidity in Dulbecco's modified eagle medium (Cellgro, Herndon, VA) supplemented with 10% (v/v) heat inactivated fetal bovine serum (BioWhittaker, Walkersville, MD), 2 mM L-glutamine and 100 units/ml Penicillin and 1000 ug/ml Streptomycin (Invitrogen). 9L and RT2 cells were transfected with the plasmids pEGFP-N2 (Clontech) or pHSP72 using Lipofectamine (Invitrogen) according to the manufacturers protocol followed by selection in 0.2 mg/ml G418 (Invitrogen). 293 cells were obtained from American Type Culture Collection (ATCC, Manassas, VA; CRL-1573) were used for virus propagation and purification.

### Plasmid Creation

pHSP72 was created by ligation of the DNA Polymerase blunt ended 2.3 KB Bam H1-Hind III fragment from plasmid pH2.3 (ATCC) into the blunt ended Xho1-XbaIA vector pEGFP-N2.

### Adenovirus

Creation of ONYX-015 has previously been described[6]. The virus was propagated on 293 cells and purified by cesium chloride gradient followed by dialysis. Virus was stored at -80°C until use. Titer was determined by tissue culture infectious dose-50 method (TCID-50). Briefly, 293 cells were plated at 10^4 ^cells/well in 96 well flat-bottomed tissue culture plates. Virus titer from combined supernatant and freeze-thawed cell lysate was determined by serial 10-fold dilution into different rows of the titer plate. After 10 days, wells were scored for cytopathic effect. The titer was calculated using the formula T = 10^1+d(S-0.5) ^where d is the Log 10 of the dilution and S is the sum of the ratios of positive wells in each row. A more lengthy description of this method is provided in the adenovirus application manual at .

### Adenovirus Infection

Cells were infected when 50% confluent with the dose of adenovirus given in the appropriate table. The infection was performed in serum free DMEM media for 90 minutes at 37°C and 5% CO_2_. Cells were subsequently washed with phosphate buffered saline and then cultured in complete medium.

### Cell Viability Determination

Cell viability was determined by the standard Trypan-blue exclusion test. Cells were washed with PBS and then lifted from tissue culture plates with Trypsin-EDTA solution (Invitrogen). Cells were stained with 0.2% Trypan blue solution for 5 minutes and then counted on a hemacytometer.

## Authors' contributions

JM performed all of the experiments outlined in this study. JAK independently replicated results for validity. JAK and MRS contributed equally to the molecular cloning of the plasmids and adenovirus propagation and purification. All authors participated in maintenance of cells in culture. The hypothesis, study design and statistical analysis were all performed by MRS. MRS drafted the entire manuscript with editing and approval by all of the authors.

## References

[B1] Walther W, Stein U (2000). Viral vectors for gene transfer: a review of their use in the treatment of human diseases. Drugs.

[B2] Wildner O (2001). Oncolytic viruses as therapeutic agents. Ann Med.

[B3] Barker DD, Berk AJ (1987). Adenovirus proteins from both E1B reading frames are required for transformation of rodent cells by viral infection and DNA transfection. Virology.

[B4] Kirn D (2001). Clinical research results with dl1520 (Onyx-015), a replication-selective adenovirus for the treatment of cancer: what have we learned?. Gene Ther.

[B5] Kirn D (2001). Oncolytic virotherapy for cancer with the adenovirus dl1520 (Onyx-015): results of phase I and II trials. Expert Opin Biol Ther.

[B6] Cohen EE, Rudin CM (2001). ONYX-015. Onyx Pharmaceuticals. Curr Opin Investig Drugs.

[B7] Putzer BM, Stiewe T, Parssanedjad K, Rega S, Esche H (2000). E1A is sufficient by itself to induce apoptosis independent of p53 and other adenoviral gene products. Cell Death Differ.

[B8] Grand RJ, Byrd PJ, Grabham PW, Gregory CD, Huen DS, Merrick RM, Young LS, Gallimore PH (1989). The expression of the retinoblastoma gene product Rb1 in primary and adenovirus-transformed human cells. Oncogene.

[B9] Ridgway PJ, Hall AR, Myers CJ, Braithwaite AW (1997). p53/E1b58kDa complex regulates adenovirus replication. Virology.

[B10] Roth J, Dobbelstein M (2003). Interaction of p53 with the adenovirus E1B-55 kDa protein. Methods Mol Biol.

[B11] Hall AR, Dix BR, O'Carroll SJ, Braithwaite AW (1998). p53-dependent cell death/apoptosis is required for a productive adenovirus infection. Nat Med.

[B12] Bischoff JR, Kirn DH, Williams A, Heise C, Horn S, Muna M, Ng L, Nye JA, Sampson-Johannes A, Fattaey A, McCormick F (1996). An adenovirus mutant that replicates selectively in p53-deficient human tumor cells. Science.

[B13] Edwards SJ, Dix BR, Myers CJ, Dobson-Le D, Huschtscha L, Hibma M, Royds J, Braithwaite AW (2002). Evidence that replication of the antitumor adenovirus ONYX-015 is not controlled by the p53 and p14(ARF) tumor suppressor genes. J Virol.

[B14] Glotzer JB, Saltik M, Chiocca S, Michou AI, Moseley P, Cotten M (2000). Activation of heat-shock response by an adenovirus is essential for virus replication. Nature.

[B15] Haviv YS, Blackwell JL, Li H, Wang M, Lei X, Curiel DT (2001). Heat shock and heat shock protein 70i enhance the oncolytic effect of replicative adenovirus. Cancer Res.

[B16] Simon MC, Kitchener K, Kao HT, Hickey E, Weber L, Voellmy R, Heintz N, Nevins JR (1987). Selective induction of human heat shock gene transcription by the adenovirus E1A gene products, including the 12S E1A product. Mol Cell Biol.

[B17] Li Z, Zhao X, Wei Y (2004). Regulation of apoptotic signal transduction pathways by the heat shock proteins. Sci China C Life Sci.

[B18] Kao HT, Capasso O, Heintz N, Nevins JR (1985). Cell cycle control of the human HSP70 gene: implications for the role of a cellular E1A-like function. Mol Cell Biol.

[B19] Matsuzaki A, Shiroki K, Kimura G (1987). Induction of cellular DNA synthesis by adenovirus type 12 in a set of temperature-sensitive mutants of rat 3Y1 fibroblasts blocked in G1 phase. Virology.

[B20] White E, Spector D, Welch W (1988). Differential distribution of the adenovirus E1A proteins and colocalization of E1A with the 70-kilodalton cellular heat shock protein in infected cells. J Virol.

[B21] Ganly I, Mautner V, Balmain A (2000). Productive replication of human adenoviruses in mouse epidermal cells. J Virol.

[B22] Hemmi S, Geertsen R, Mezzacasa A, Peter I, Dummer R (1998). The presence of human coxsackievirus and adenovirus receptor is associated with efficient adenovirus-mediated transgene expression in human melanoma cell cultures. Hum Gene Ther.

[B23] Jaattela M, Wissing D, Kokholm K, Kallunki T, Egeblad M (1998). Hsp70 exerts its anti-apoptotic function downstream of caspase-3-like proteases. Embo J.

[B24] Imperiale MJ, Kao HT, Feldman LT, Nevins JR, Strickland S (1984). Common control of the heat shock gene and early adenovirus genes: evidence for a cellular E1A-like activity. Mol Cell Biol.

[B25] Niewiarowska J, D'Halluin JC, Belin MT (1992). Adenovirus capsid proteins interact with HSP70 proteins after penetration in human or rodent cells. Exp Cell Res.

[B26] Macejak DG, Luftig RB (1991). Association of HSP70 with the adenovirus type 5 fiber protein in infected HEp-2 cells. Virology.

[B27] Wust P, Hildebrandt B, Sreenivasa G, Rau B, Gellermann J, Riess H, Felix R, Schlag PM (2002). Hyperthermia in combined treatment of cancer. Lancet Oncol.

[B28] Benda P, Someda K, Messer J, Sweet WH (1971). Morphological and immunochemical studies of rat glial tumors and clonal strains propagated in culture. J Neurosurg.

[B29] Barth RF (1998). Rat brain tumor models in experimental neuro-oncology: the 9L, C6, T9, F98, RG2 (D74), RT-2 and CNS-1 gliomas. J Neurooncol.

